# Abdominal Surgical Emergencies in Patients with Hematological Disorders: An Exacting Experience for Surgeons

**DOI:** 10.7759/cureus.4017

**Published:** 2019-02-05

**Authors:** Abinaya R Nadarajan, Gigi Varghese

**Affiliations:** 1 General Surgery, Christian Medical College Hospital, Vellore, IND

**Keywords:** hematological disorders, glanzmann’s thrombasthenia, paroxysmal nocturnal hemoglobinuria

## Abstract

Management of surgical emergencies in patients with underlying hematological disorder is challenging due to increased incidence of peri-operative morbidity. We report two cases of abdominal surgical emergencies with pre-existing hematological conditions. The first case report is that of a patient diagnosed with pelvic abscess in a previously diagnosed case of Glanzmann’s thrombasthenia and the second is a case of intestinal infarction previously diagnosed with paroxysmal nocturnal hemoglobinuria (PNH).

## Introduction

Glanzmann’s thrombasthenia is a rare genetic platelet disorder with autosomal recessive inheritance. It is a qualitative disorder due to abnormalities of glycoprotein (GP) IIb-IIIa membrane complex which helps in platelet aggregation [1]. It is more common in females and manifests as mucocutaneous bleeding, epistaxis, and menorrhagia [1-2]. The precipitating factors are trauma or surgery as spontaneous bleeding is uncommon. Management of these patients is extremely difficult as they need continuous assessment of bleeding parameters and platelet transfusion for any invasive procedure to control hemorrhage. Patients with Glanzmann’s thrombasthenia can present with spontaneous overt gastrointestinal bleeding [3].

In the other end of the spectrum is paroxysmal nocturnal hemoglobinuria (PNH), which has an increased thrombotic tendency. It is a rare acquired disorder characterized by hemolytic anemia, thrombosis, and impaired bone marrow function [4]. This is due to the mutation of phosphatidylinositol glycan class A (PIG-A) gene in the bone marrow stem cells and they are deficient in glycosylphosphatidylinositol (GPI) anchor proteins. Flow cytometry is the recommended investigation to diagnose PNH. Thrombosis occurs in 40% of the patients and most commonly seen in the venous system [4]. The pathogenesis of thrombophilia in PNH is speculative. The most common sites of thrombosis are hepatic, mesenteric veins, portal, and cerebral veins. Venous thrombosis in cerebral and splanchnic circulation is a major cause of morbidity and mortality in PNH patients [5]. The incidence of mesenteric vein thrombosis leading to bowel ischemia is 3% to 8% [6]. Bowel ischemia and infarction in patients with PNH undergoing major bowel resection as an emergency surgical intervention have been reported in the literature [7].

## Case presentation

Case 1

**Figure 1:**
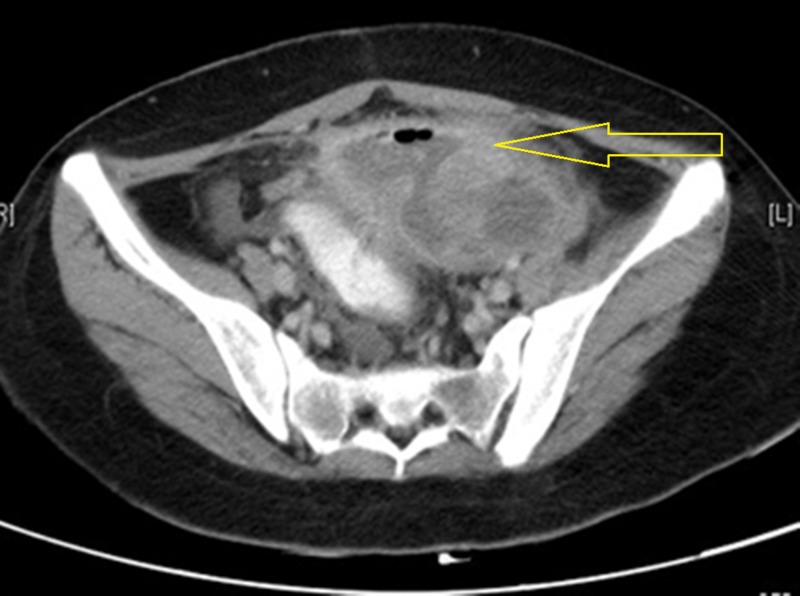
Contrast-enhanced computed tomography (CECT) of the abdomen and pelvis showing multiloculated pelvic collection with significant sigmoid wall thickening indicating sigmoid perforation

An 18-year-old female who was previously diagnosed with Glanzmann’s thrombasthenia had undergone laparoscopic aspiration of the ovarian cyst three weeks back at another center. She was on synthetic progesterone and tranexamic acid which is an antifibrinolytic agent for menorrhagia. She presented to us with lower abdominal pain, fever, loose stools, and intermittent rectal bleeding. Her blood picture revealed low hemoglobin of 8 gm/dl, increased white cell count of 14000 cells per cubic millimeter, normal platelet count, and normal prothrombin time and partial thromboplastin time. Contrast-enhanced computed tomography (CECT) of the abdomen showed pelvic collection with an air pocket and thickened sigmoid colon (Figure 1). Colonoscopy was performed as she had persistent bleeding per rectum with low hemoglobin and it showed multiple colonic mucosal hemorrhages and a fistulous opening in the sigmoid colon, probably an iatrogenic perforation which happened during prior laparoscopic cyst aspiration. She was planned for computed tomography (CT) guided drainage of the pelvic collection under platelet cover as she was hemodynamically stable and the CT showed only localised collection. Since source control with radiology-guided drainage was inadequate and the patient had persistent fever with loose stools, she was taken up for laparotomy and pelvic abscess drainage. A sigmoid colectomy with proximal end colostomy was also performed as there was a sigmoid perforation which resulted in pelvic abscess and dense adhesions. She received single donor platelets and irradiated packed red blood cells during surgery. In the postoperative period, she had a surgical site hematoma and her hemoglobin dropped to 5.6 gm/dl. Irradiated packed red cells and human leukocyte antigen (HLA) matched single donor platelets were transfused. Thromboelastography (TEG) was performed pre- and post-platelet transfusion to assess the response as she had previously received platelet transfusions and the risk of alloimmunisation was high. Following transfusion, her hemoglobin was stable and there was no further active bleeding. She had a prolonged hospital stay and delayed postoperative recovery due to protracted ileus, hematoma, and superficial surgical site infection. Her central line and skin staples were removed under platelet cover as per the instructions of the hematology team.

Case 2

A 26-year-old male who was previously diagnosed to have PNH and had been in treatment with steroids and anti-thymocyte globulin, presented with acute abdominal pain and fever. He presented with signs of peritonitis. His blood picture revealed low hemoglobin of 8 gm/dl and low white cell count of 2900 cells per cubic millimeter and normal platelet count. He was taken up for emergency laparotomy as he was septic with peritonitis after adequate blood transfusion. He was found to have jejunal intussusception and proximal ileal segmental infarction with thrombosed mesenteric veins. This segment of small bowel was resected and viable ileal ends were anastomosed. In the postoperative period, he was started on parenteral anticoagulant for mesenteric vein thrombosis probably due to PNH. He was closely monitored in the intensive care unit and was started on total parenteral nutrition, as there was a delay in starting the enteral feed due to prolonged ileus. Histopathological examination of resected bowel showed an extensive transmural hemorrhagic infarction.

## Discussion

Elective and emergency abdominal surgery in patients with bleeding and thrombotic syndromes carries high morbidity and mortality. It is important for surgeons to anticipate the perioperative management of anticipated complications in order to achieve optimal goals. Glanzmann’s thrombasthenia is a rare autosomal recessive qualitative platelet disorder due to deficiency or dysfunction of platelet membrane glycoprotein GPIIb/IIIa complex [1-2,8]. This glycoprotein complex plays a major role in platelet adhesion and aggregation. These patients are at risk of mucocutaneous bleeding or bleeding during minor or major surgical procedures. Platelet transfusion is the standard treatment to prevent major bleeding during or after surgery. Repeated platelet transfusion can result in alloimmunisation and patients with alloantibody to GPIIb/IIIa will become refractory to future platelet transfusion treatments. For this reason, single donor HLA matched platelet transfusions are preferred to delay or prevent the alloimmunisation [8]. Patients diagnosed to have Glanzmann's thrombasthenia who were refractory to platelet transfusions can be treated with recombinant human activated factor VIIa [8]. These patients should be evaluated preoperatively for alloantibody to glycoprotein receptor. It is important to check the availability of blood products before planning any invasive procedure. Patients with Glanzmann’s thrombasthenia undergoing surgery need appropriate evaluation and a multidisciplinary approach including a surgeon, hematologist, and transfusion physician is required to reduce the blood loss during and after surgery. This patient received packed red blood cells and platelets during surgery and in the postoperative period with close observation of blood counts, coagulation parameters as per the instructions by the hematology team.

PNH is a rare clonal hematopoietic stem cell disorder, where the cell acquires a mutation in PIG-A gene [4,7,9]. PNH stem cells are deficient in GPI anchor proteins and other proteins, which play a role in complement cascade. Due to this, complement-mediated intravascular and extravascular hemolysis of PNH cells occurs. The pathogenesis of thrombosis in PNH is still not clear but it is attributed to intravascular hemolysis [7,9]. PNH manifests as bone marrow failure, hemolytic anemia, and thrombosis. Several cases of bowel ischemia due to mesenteric vein thrombosis have been reported in the literature [6-7]. 

Flow cytometry aid in the diagnosis of PNH along with clinical assessment [4]. Patients presenting with abdominal pain in PNH should always raise a suspicion of mesenteric vein thrombosis. In case of acute abdomen with a suspicion of intestinal infarction, emergency laparotomy is warranted. Thrombosis is life threatening in PNH, as it is progressive and refractory to anticoagulation therapy. Lifelong anticoagulation is still an area of debate in the treatment of PNH with thrombotic event [5].

Eculizumab is a humanized monoclonal antibody which blocks the complement cascade and reduces intravascular hemolysis and thrombosis. Eculizumab is indicated in transfusion-dependent hemolysis and PNH related complications like thrombosis and renal failure [4,9-10]. Bone marrow transplant is the only curative therapy for PNH [4]. Our patient with PNH had intestinal infarction and had undergone ileal resection and anastomosis. He was started on anticoagulant therapy with frequent monitoring of blood counts and coagulation parameters. He was planned for bone marrow transplant in future.

## Conclusions

Underlying disorders of clotting mechanism can significantly complicate surgical emergencies requiring a multidisciplinary approach. A preoperative algorithm has to be designed and individualized for patients with such abnormalities anticipating unique intraoperative and postoperative complications.
